# Treatment outcome of oral appliance in patients with REM-related obstructive sleep apnea

**DOI:** 10.1007/s11325-019-01966-5

**Published:** 2019-11-25

**Authors:** Yoshitomo Nishio, Tetsuro Hoshino, Kenta Murotani, Akifumi Furuhashi, Masayo Baku, Ryujiro Sasanabe, Yoshiaki Kazaoka, Toshiaki Shiomi

**Affiliations:** 1 Department of Oral and Maxillofacial Surgery, Aichi Medical University Hospital, 1-1 Yazakokarimata, Nagakute, Aichi 4801195 Japan; 2 Department of Sleep Medicine and Sleep Disorders Center, Aichi Medical University Hospital, 1-1 Yazakokarimata, Nagakute, Aichi 4801195 Japan; 3 Biostatistics Center, Graduate School of Medicine, Kurume University, 67 Asahimachi, Kurume, Fukuoka, 8300011 Japan

**Keywords:** Obstructive sleep apnea, REM-related obstructive sleep apnea, Oral appliance, Body mass index

## Abstract

**Purpose:**

Oral appliances (OA) are used to treat patients with obstructive sleep apnea (OSA). The purpose of this study is to evaluate the efficacy of OA treatment in patients with rapid eye movement (REM)–related OSA.

**Methods:**

Forty-six patients with REM-related OSA and 107 with non-stage-specific OSA were prescribed OA treatment after diagnosis by polysomnography (PSG) and a follow-up sleep test by PSG was conducted. Efficacy and treatment outcome predictors were evaluated according to the following criteria for treatment success: #1, reduction of the apnea-hypopnea index (AHI) to less than 5 and > 50% compared with baseline; #2, AHI reduction to less than 10 and > 50% compared with baseline; and #3, > 50% AHI reduction compared with baseline.

**Results:**

Success rates according to criteria #1, #2, and #3 were 45.7%, 50.0%, and 50.0% in REM-related OSA and 36.4%, 52.3%, and 63.6% in non-stage-specific OSA, respectively. No significant differences in success rate were found between the two groups. In multivariate logistic regression analysis with each criterion as the response variable, only BMI was extracted as a significant predictor. The BMI cutoff values defined based on the maximum Youden index according to the three criteria were 26.2 kg/m^2^, 25.6 kg/m^2^, and 26.2 kg/m^2^, respectively.

**Conclusions:**

No significant differences in success rate of OA treatment were found between REM-related OSA and non-stage-specific OSA. BMI has greater impact on treatment outcome of OA in patients with REM-related OSA.

## Introduction

Obstructive sleep apnea (OSA), characterized by repetitive respiratory events including apnea and hypopnea, is due to total or partial collapse of the upper airways during sleep, and affects 9 to 38% of the general adult population [1]. Untreated OSA is associated with daytime symptoms, various comorbidities, and mortality [2]. Although continuous positive airway pressure (CPAP) is clearly a highly effective treatment option, various alternative treatment options are available, such as oral appliances, upper airway surgery, and hypoglossal nerve stimulation, with sufficient evidence supporting their use in selected patient populations [3]. Thus sleep medicine for OSA is moving into the era of personalized treatment.

Rapid eye movement (REM)–related OSA, a highly prevalent subtype of OSA affecting 13 to 36%of patients, is characterized by apnea and hypopnea events predominantly or exclusively occurring during REM sleep [4, 5]. Although the pathophysiology of REM-related OSA is still unclear and its overall severity as defined by the apnea and hypopnea index (AHI) tends to be mild to moderate, it should be evaluated separately from non-stage-specific OSA, because a recent cohort study indicated that REM-related OSA is independently associated with important cardiovascular risk factors such as hypertension, metabolic syndrome, and diabetes [6]. Therefore, appropriate management of these patients is crucial.

However, it is difficult to determine the appropriate course of management due to the lack of clinical data regarding the aforementioned treatment options for REM-related OSA. Moreover, two recent clinical studies have indicated that it is difficult for patients with REM-related OSA to achieve good adherence, and these patients are occasionally intolerant to continuous positive airway pressure (CPAP) therapy, which is the standard OSA treatment option [7, 8]. Hence, we consider it an urgent challenge to collect clinical data for each treatment option for this specific type of OSA.

The use of oral appliances (OA) in OSA treatment, and specifically of mandibular advancement devices, which prevent upper airway collapse by protruding the mandible forward and altering the tongue position, is supported by strong evidence [9]. Clinical guidelines recommend OA treatment for patients with mild to moderate OSA and for those with severe OSA who are intolerant to CPAP therapy or refuse it [10]. Therefore, sleep clinicians have many opportunities to prescribe OA for patients with REM-related OSA. However, only one study reported the efficacy of OA in REM-related OSA [11], so that the level of evidence about the efficacy of this treatment is low.

Cephalometric analysis is highly recommended in patients with OSA as one of the most important tools for diagnosis and treatment planning [12]. In addition, several studies reported that specific cephalometric measurements predict OA treatment outcome [13]. However, only one study evaluated the craniofacial characteristics of the patients with REM-related OSA using cephalometric measurements [14]. Moreover, no study evaluated cephalometric measurements as predictors of OA treatment success in patients with REM-related OSA.

The aim of this study was to evaluate the outcome of OA treatment and clarify its predictors in patients with REM-related OSA.

## Materials and methods

Single-center retrospective observational study assessed patients who were prescribed OA after a diagnosis of OSA by polysomnography (PSG) at the Department of Sleep Medicine of the Aichi Medical University Hospital from January 2007 to December 2018. At the time of their first visit to the Department of Oral and Maxillofacial Surgery, all patients underwent craniofacial evaluation by cephalometry.

### Nocturnal polysomnography

Diagnostic and follow-up nocturnal PSG was performed using the Alice 4 or 5 system (Respironics, Inc., Murrysville, PA, USA). The following biological variables were continuously monitored: electrocardiogram, electroencephalogram, chin and anterior tibialis electromyogram, bilateral electro-oculogram, airflow measurement using a nasal thermistor, arterial oxygen saturation, respiratory effort measured by thoracic and abdominal inductive plethysmography bands, body position, and snoring. Respiratory events, including apnea and hypopnea, and other PSG parameter, were scored manually by sleep technicians according to the 2007 guidelines of the American Academy of Sleep Medicine (AASM) [15]. The apnea-hypopnea index (AHI) was defined as the average number of apnea and hypopnea events per hour of sleep, and OSA was defined as AHI ≥ 5, following the International Classification of Sleep Disorder (ICSD)-2 criteria [16].

We defined REM-related OSA as an overall AHI ≥ 5, a ratio of AHI during REM sleep (AHI_REM_)/AHI during NREM sleep (AHI_NREM_) ≥ 2, and AHI_NREM_ < 15, which is the definition most widely reported in the literature [4, 17–19].

### Oral appliance

The OA was designed to protrude the mandible to maintain upper airway patency. A custom-made monobloc mandibular advancement oral appliance made from a 2.0-mm polyethylene plate (Erkodur; Erkodent Inc.; Pfalzgrafenweiler, Germany) was prescribed for all participants. The construction bite was registered at 70% of the maximum mandibular protruded position, while its vertical position was set at the minimum occlusal elevation that allowed for mandibular advancement [20].

Treatment outcome for OA was evaluated according to the following alternative criteria for success: #1, reduction of the AHI to a value < 5 and > 50% AHI reduction compared with baseline, the strictest criterion; #2, reduction of the AHI to a value < 10 and > 50% AHI reduction compared with baseline, the most frequently used criterion in the literature; and #3, > 50% AHI reduction compared with baseline [21, 22].

### Cephalometric evaluation

**Fig. 1:**
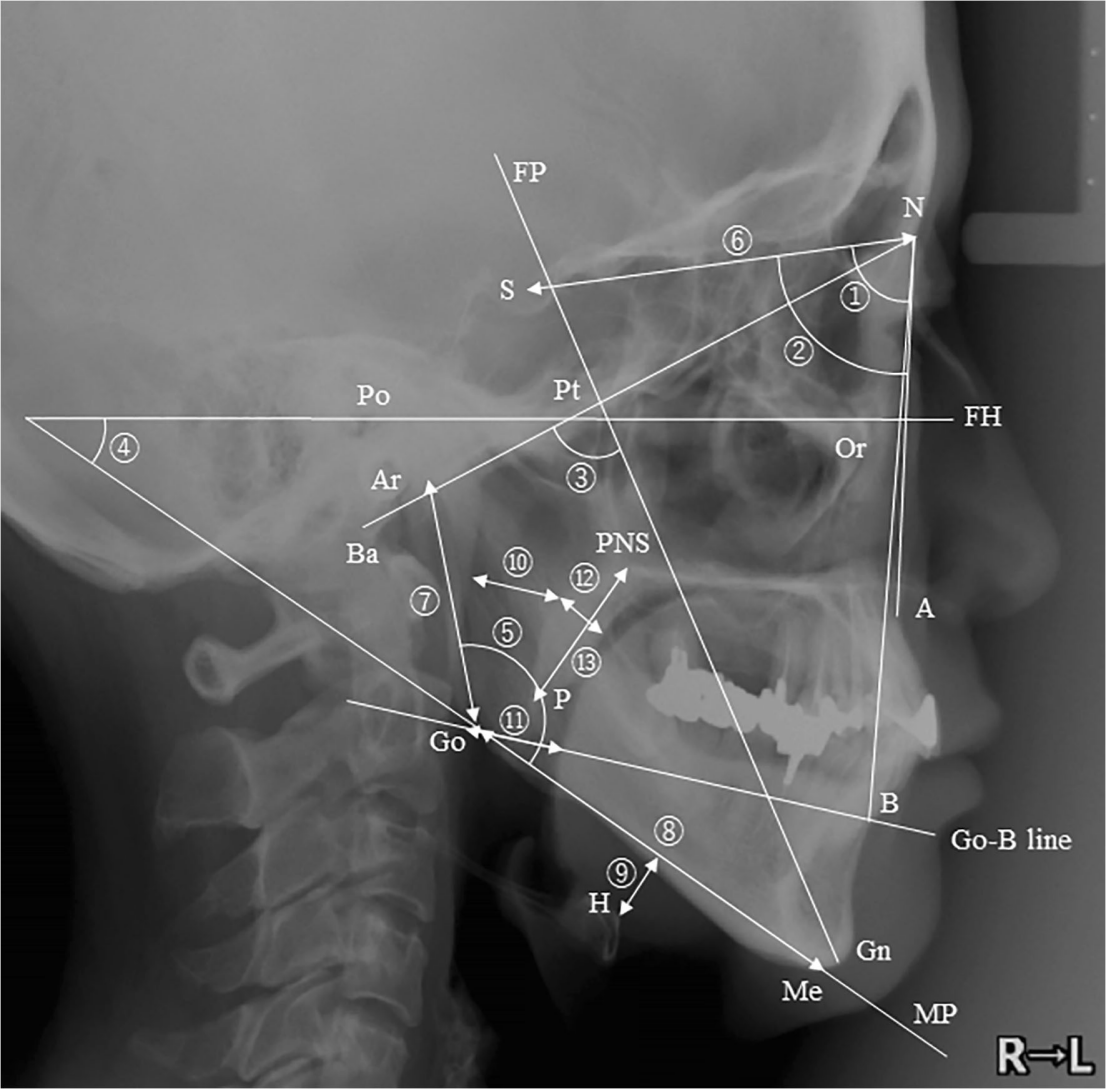
Cephalometric landmarks and reference planes: N (Nasion), S (sella), Ba (basion), Po (porion), Ar (articulare), Or (orbitale), Pt (pterygoid point), PNS (posterior nasal spine), A (point-A), B (point-B), Me (menton), Gn (gnathion), Go (gonion), and H (hyoid). FH, Frankfort horizontal plane; MP, mandibular plane; FP, facial plane. ① SNA (angle formed between sella, nasion, and point A), ② SNB (angle formed between sella, nasion, and point B), ③ facial axis (angle formed between FP and N-Ba), ④ mandibular plane angle (Angle formed between FH plane and mandibular plane), ⑤ gonion angle (angle formed between mandibular plane and Ar-Go). ⑥ S-N (linear distance between S and N), ⑦ Ar-Go (linear distance between Ar and Go), ⑧ Go-Me (linear distance between Go and Me), ⑨ MP-H (linear distance between the mandibular plane and H). ⑩ SPAS (width of the airway behind the soft palate along the line parallel to the Go-B line), ⑪ IAS (width of the airway along the Go-B line), ⑫ UD (soft palate thickness), and ⑬ UL (soft palate length)

Lateral cephalometric radiography was performed for all the participants in the upright position with the Frankfort horizontal plane parallel to the ground. The film was taken while holding their breath at the end of the inspiratory phase with closed lips and teeth in centric occlusion. A single investigator, blinded to the demographic and polysomnographic status of the participants, traced all cephalometric radiographs. The cephalometric landmarks and measurements used in this study are detailed in Fig. 1.

### Statistical analysis

All continuous variables were expressed as median (25th–75th percentile). The Student’s *t* test was used for normally distributed data, and the Mann-Whitney *U* test for non-normally distributed data. Normality was assessed using the Shapiro-Wilk test. Categorical variables were expressed as numbers (percentages) and compared using Fisher’s exact test. Predictive factors of OA treatment success were evaluated by multivariate logistic regression using the backward selection method, including all variables assessed in the study. The predictive ability was assessed by receiver operating characteristic (ROC) analysis using the area under curve (AUC). *P* values < 0.05 were considered statistically significant. Statistical analyses were performed using the SPSS software program for Windows, version 25.0 (SPSS Inc., Chicago, IL, USA) and the R statistical package (version 3.5.0, R Foundation for Statistical Computing).

## Results

**Fig. 2:**
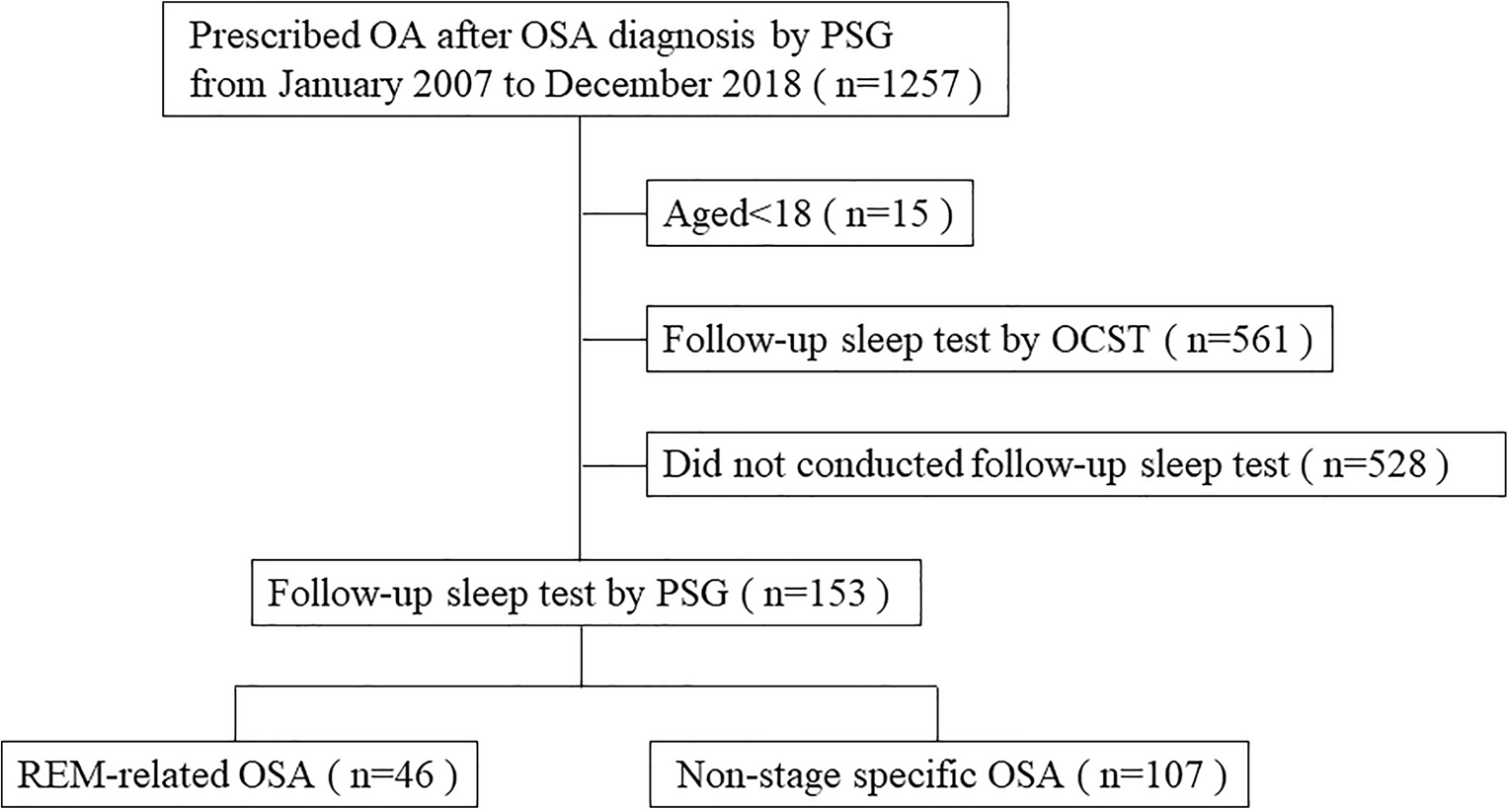
Flow diagram of the study. OA, oral appliance; OSA, obstructive sleep apnea; PSG, polysomnography; OCST, out of center sleep test; REM, rapid eye movement

We assessed 1257 patients. Fifteen patients aged < 18 years, 561 patients who were followed up by out of center sleep testing (OCST), and 528 patients who did not undergo follow-up sleep testing were excluded. Ultimately, 153 adult OSA patients who underwent follow-up sleep testing by PSG were enrolled in the study (Fig. 2).

Out of 153 patients, 46 were REM-related OSA and 107 were non-stage-specific OSA. Table 1 shows the demographic and polysomnographic findings in patients with REM-related OSA and non-stage-specific OSA. Significant differences were found in sleep efficiency [87.1% (79.5–94.6) vs. 82.4% (70.8–91.7); *p* = 0.013], REM/total sleep time (TST) [18.7% (13.4–23.3) vs. 15.0% (11.9–15.0), *p* = 0.021], number of patients with 5 ≤ AHI < 15 [36 (78.3%) vs. 30 (28.0%), *p* < 0.001], number of patients with AHI ≥ 30 [0 (0%) vs. 36 (33.6%), *p* < 0.001], and percentage of time spent at SaO_2_ below 90% (CT90) [0.7% (0.1–0.7) vs. 1.3% (0.2–5.0), *p* = 0.039].

**Table 1:** Demographic and polysomnographic findings in the patients with REM-related OSA and non-stage-specific OSA

	REM-related OSA (*n* = 46)	Non-stage-specific OSA (*n* = 107)	*p* value
Age	61.0 (55.0–66.8)	64.0 (51.0–73.0)	0.523
Sex, male (%)	30 (65.2)	80 (74.8)	0.248
BMI (kg m^2^)	24.3 (22.5–26.1)	24.3 (21.0–26.7)	0.775
ESS score	8 (4–13)	8 (5–13)	0.892
Sleep efficiency (%)	87.1 (79.5–94.6)	82.4 (70.8–91.7)	0.013*
REM latency (min)	92.0 (70.5–143.0)	109.0 (76.3–183.0)	0.139
REM/TST (%)	18.7 (13.4–23.3)	15.0 (11.9–15.0)	0.021*
5 ≤ AHI < 15 (%)	36 (78.3)	30 (28.0)	< 0.001*
15 ≤ AHI < 30 (%)	10 (21.7)	41 (38.3)	0.061
30 ≤ AHI (%)	0 (0)	36 (33.6)	< 0.001*
AHI_REM_ (/h)	32.3 (22.9–42.5)	21.8 (8.6–38.3)	0.003*
AHI_NREM_ (/h)	7.0 (4.8–10.0)	21.9 (13.8–39.3)	< 0.001*
minSpO2 (%)	83.5 (80.0–87.3)	81.0 (76.0–86.0)	0.148
CT90 (%)	0.7 (0.1–1.7)	1.3 (0.2–5.0)	0.039*

*REM* rapid eye movement, *OSA* obstructive sleep apnea, *BMI* body mass index, *ESS* Epworth Sleepiness Scale, *TST* total sleep time, *AHI* apnea and hypopnea index, *AHI*_*REM*_ apnea and hypopnea index during REM sleep, *AHI*_*NREM*_ apnea and hypopnea index during NREM sleep, *CT90* cumulative percentage of time spent at saturation below 90%

*p < 0.05 when comparing REM-related OSA with non-stage-specific OSA

Table 2 shows the cephalometric findings in patients with REM-related OSA and non-stage-specific OSA. Significant differences were found in linear distance between articulare and gonion (Ar-Go) [54.9 mm (48.4–59.4) vs. 57.2 mm (52.5–61.3), *p* = 0.045] and between mandibular plane and hyoid distance (MP-H) [17.4 mm (11.8–19.8) vs. 18.2 mm (14.5–23.7), *p* = 0.036]. No significant differences were found in angular measurements.

**Table 2:** Cephalometric findings in REM-related OSA and non-stage-specific OSA patients

	REM-related OSA (*n* = 46)	Non-stage-specific OSA (*n* = 107)	*p* value
SNA (°)	81.6 (79.6–84)	82.3 (80.2–84.3)	0.374
SNB (°)	77.7 (75.8–80.7)	78.7 (75.7–81.2)	0.632
Facial axis (°)	81.5 (78.2–84.3)	80.9 (77.7–84.1)	0.897
Mandibular plane angle (°)	30.7 (27.4–34.9)	28.6 (24.6–33.7)	0.062
Gonion angle (°)	119.2 (114.2–124.3)	119.6 (114.8–123.6)	0.946
S-N (mm)	70.9 (67.6–73.2)	70.9 (69.1–73.3)	0.422
Ar-Go (mm)	54.9 (48.4–59.4)	57.2 (52.5–61.3)	0.045*
Go-Me (mm)	73.9 (71.1–78.3)	74.8 (72.1–79.0)	0.426
MP-H (mm)	17.4 (11.8–19.8)	18.2 (14.5–23.7)	0.036*
SPAS (mm)	9.7 (7.5–12.2)	9.8 (7.2–12.1)	0.997
IAS (mm)	13.1 (10.8–16.5)	11.7 (9.4–15.9)	0.145
UD (mm)	27.4 (9.9–36.9)	13.8 (10.3–38.8)	0.797
UL (mm)	14.1 (10.5–39)	34.4 (10.4–42.4)	0.189

*REM* rapid eye movement, *OSA* obstructive sleep apnea. See also Fig. 2 for the definition of the cephalometric parameters

*p < 0.05 when comparing REM-related OSA with non-stage-specific OSA

Table 3 shows the success rate according to each criterion in REM-related OSA and non-stage-specific OSA. The success rates according to criteria #1, #2, and #3 were 45.7%, 50.0%, and 50.0% in REM-related OSA, and 36.4%, 52.3%, and 63.6% in non-stage-specific OSA, respectively. No significant differences in success rates were found between the two groups.

**Table 3:** Success rate for each criterion in REM-related OSA and non-stage-specific OSA patients

	Total (*n* = 153)	REM-related OSA (*n* = 46)	Non-stage-specific OSA (*n* = 107)	*p* value
Criterion #1 (%)	60 (39.2%)	21 (45.7%)	39 (36.4%)	0.368
Criterion #2 (%)	79 (51.6%)	23 (50.0%)	56 (52.3%)	0.861
Criterion #3 (%)	79 (51.6%)	23 (50.0%)	68 (63.6%)	0.151

*REM* rapid eye movement, *OSA* obstructive sleep apnea

Criterion #1: reduction of AHI to a value < 5 and > 50% AHI reduction compared with baseline; Criterion #2: reduction of AHI to a value < 10 and > 50% AHI reduction compared with baseline; Criterion #3: > 50% AHI reduction compared with baseline

Table 4 shows the comparison of PSG findings, before and after OA treatment, in REM-related OSA and non-stage-specific OSA. The hypopnea index (HI) during REM sleep had no significant difference in both groups [REM-related OSA: 12.1 (3.7–23.1) vs. 5.2 (2.5–17.0), *p* = 0.183; non-stage-specific OSA: 6.7 (1.3–15.2) vs. 6.5 (1.4–17.9), *p* = 0.550].

**Table 4:** Comparison of PSG findings before and after OA treatment in REM-related OSA and non-stage-specific OSA

	Before	After	*p* value
REM-related OSA (*n* = 46)			
AHI_REM_ (/h)	32.3 (22.9–42.5)	12.1 (3.2–25.5)	< 0.001*
AHI_NREM_ (/h)	7.0 (4.8–10.0)	2.7 (0.7–5.5)	< 0.001*
AI_REM_ (/h)	16.1 (5.9–30.7)	2.2 (0.0–8.4)	< 0.001*
AI_NREM_ (/h)	2.5 (0.8–4.4)	0.1 (0.0–1.0)	< 0.001*
HI_REM_ (/h)	12.1 (3.7–23.1)	5.2 (2.5–17.0)	0.183
HI_NREM_ (/h)	6.7 (1.8–12.0)	3.8 (0.8–6.8)	0.020*
Maximum desaturation_REM_ (%)	12.5 (8.8–17.3)	8.0 (4.8–11.3)	0.002*
Maximum desaturation_NREM_ (%)	9.0 (7.0–12.0)	6.0 (4.0–10.3)	0.011*
CT90_REM_ (%)	0.4 (0.0–1.1)	0.0 (0.0–0.2)	0.003*
CT90_NREM_ (%)	0.0 (0.0–0.2)	0.0 (0.0–0.0)	0.032*
Non-stage-specific OSA (*n* = 107)			
AHI_REM_ (/h)	21.8 (8.6–38.3)	11.5 (2.7–29.4)	0.006*
AHI_NREM_ (/h)	21.9 (13.8–39.3)	6.4 (1.7–13.4)	< 0.001*
AI_REM_ (/h)	10.4 (1.2–24.8)	1.5 (0.0–10.1)	< 0.001*
AI_NREM_ (/h)	10.0 (2.2–16.6)	0.5 (0.0–1.8)	< 0.001*
HI_REM_ (/h)	6.7 (1.3–15.2)	6.5 (1.4–17.9)	0.550
HI_NREM_ (/h)	10.1 (5.1–21.1)	5.9 (1.2–13.3)	< 0.001*
Maximum desaturation_REM_ (%)	14.0 (5.0–21.0)	8.0 (4.0–12.0)	< 0.001*
Maximum desaturation_NREM_ (%)	13.0 (8.0–17.0)	7.0 (5.0–10.0)	< 0.001*
CT90_REM_ (%)	0.3 (0.0–1.1)	0 (0.0–0.2)	< 0.001*
CT90_NREM_ (%)	0.2 (0.0–1.5)	0 (0.0–0.0)	< 0.001*

*REM* rapid eye movement, *NREM* non-rapid eye movement, *AHI* apnea and hypopnea index, *AI* apnea index, *HI* hypopnea index, *CT90* cumulative percentage of time spent at saturation below 90%

*p < 0.05 when comparing the PSG findings before and after OA treatment PSG findings

Table 5 shows the results of the multivariate logistic regression analysis of the treatment outcome for REM-related OSA with the backward selection method including all variables assessed in this study. In the analysis with criterion #1 as the response variable, BMI [OR, 0.730; 95% CI, 0.559–0.955; *p* = 0.022] was independently associated with treatment success. In the analysis with criterion #2 as the response variable, BMI [OR, 0.732; 95% CI, 0.562–0.953; *p* = 0.02] was independently associated with treatment success. In the analysis with criterion #3 as the response variable, BMI [OR, 0.674; 95% CI, 0.501–0.907; *p* = 0.009] was independently associated with treatment success.

**Table 5:** Multivariate logistic regression analysis for predictors of OA treatment success in the patients with REM-related OSA

	Variable	Coefficient	OR	95% CI	*p* value
Criterion #1	BMI (kg/m^2^)	− 0.314	0.730	0.559–0.955	0.022*
Criterion #2	BMI (kg/m^2^)	− 0.312	0.732	0.562–0.953	0.020*
Criterion #3	BMI (kg/m^2^)	− 0.394	0.674	0.501–0.907	0.009*
	Go-Me (mm)	− 0.107	0.898	0.795–1.016	0.087

*OR* odds ratio, *95% CI* 95% confidence interval

Criterion #1: reduction of AHI to a value < 5 and > 50% AHI reduction compared with baseline; Criterion #2: reduction of AHI to a value < 10 and > 50% AHI reduction compared with baseline; Criterion #3: > 50% AHI reduction compared with baseline

*p < 0.05 when comparing REM-related OSA with non-stage-specific OSA

**Fig. 3:**
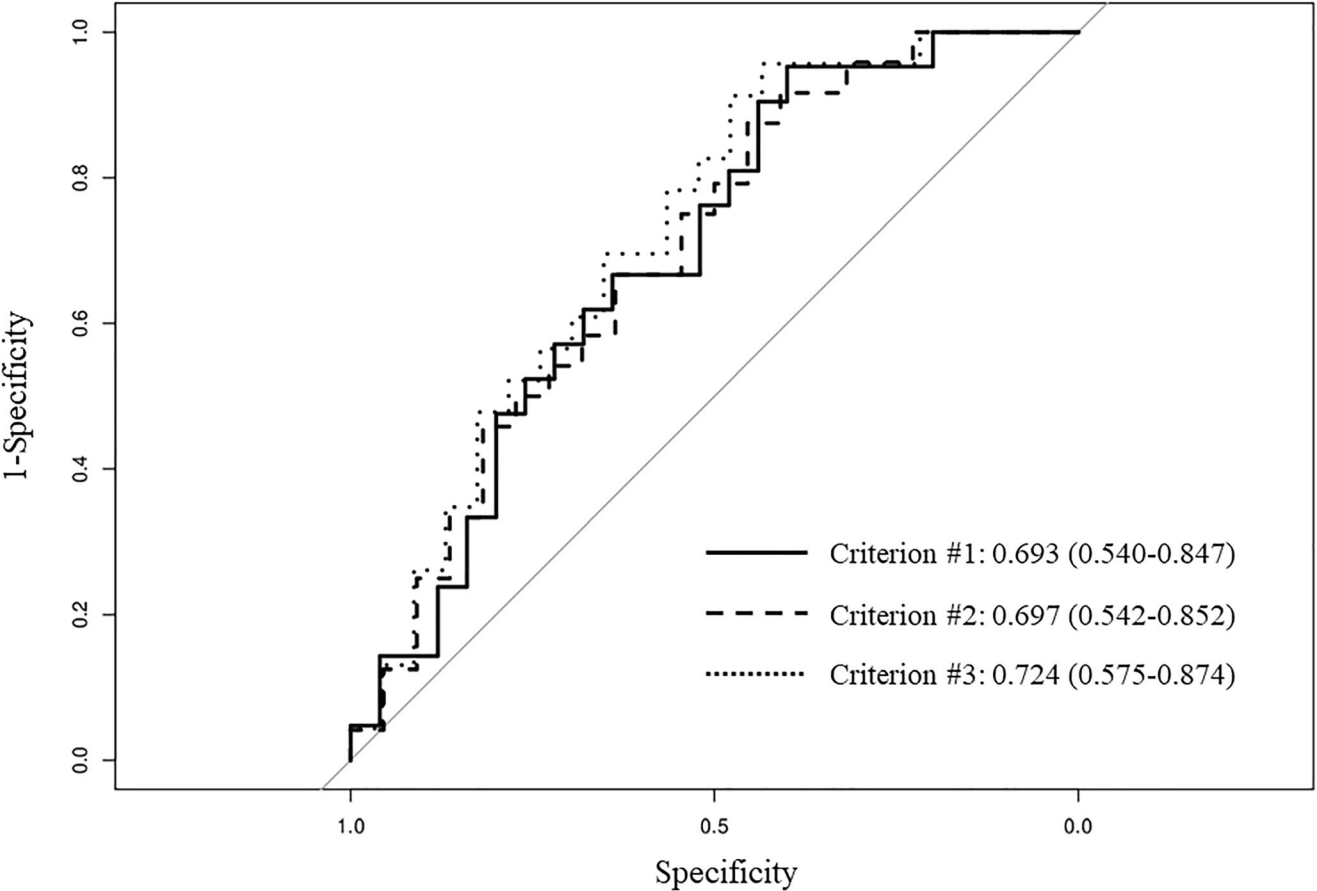
Receiver operating characteristics (ROC) curves of BMI for each treatment success criteria. The areas under the curve for BMI in criteria #1, #2, and #3 were 0.693, 0.697, and 0.724, respectively

ROC curves were plotted to evaluate the predictive ability of BMI for each treatment success criterion in Fig 3. The areas under the ROC curve for BMI as a predictor of success according to criteria #1, #2, and #3 were 0.693 (95% CI, 0.540–0.847), 0.697 (0.542–0.852), and 0.724 (0.575-0.874), respectively. The best BMI cutoff values, defined as the maximum Youden index, according to criteria #1, #2, and #3 were 26.2 kg/m^2^ [sensitivity 95.2% (95% CI, 0.857–1.000), specificity 40.0%(95% CI, 0.240–0.600)], 25.6 kg/m^2^ [sensitivity 87.5% (95% CI, 0.750–1.000), specificity 45.5% (95% CI, 0.227–0.636)], and 26.2 kg/m^2^ [sensitivity 95.7% (95% CI, 0.870–1.000), specificity 43.5% (95% CI, 0.217–0.652)], respectively.

## Discussion

To our knowledge, this is the first study evaluating predictors of treatment outcome for OA in patients with REM-related OSA, and also the first study evaluating OA treatment outcome and craniofacial characteristics in Japanese patients with REM-related OSA.

In craniofacial evaluation, REM-related OSA had significantly superior hyoid position compared with non-stage-specific OSA as measured by the distance from mandibular plane and hyoid bone (MP-H). An inferiorly displaced hyoid as measured by MP-H has been consistently associated with OSA severity [23]. Therefore, this result might reflect lower and stable AHI during NREM sleep which is major pathophysiological characteristics of REM-related OSA. In addition, none of the angular measurements, including sella–nasion–A point angle (SNA), sella–nasion–B point angle (SNB), facial axis, mandibular plane angle, and gonion angle, showed significant differences between the two groups. Only one previous study by Eun et al. evaluated craniofacial differences using 5 cephalometric measurements between the two groups, and also found no significant differences in any of the angular measurements assessed [14]. Although it is still unclear whether REM-related OSA has craniofacial characteristics different from non-stage-specific OSA, due to the limited data available, these results might indicate important aspects of the craniofacial characteristics of REM-related OSA. Further studies will be necessary to confirm our results, and will also contribute to elucidating the unknown pathophysiology of REM-related OSA.

Only the previous study by Sutherland et al. evaluated OA treatment outcomes for patients with REM-related OSA, and showed that complete response, defined by a reduction of the AHI to less than 5 events/h, which generally can be achieved by CPAP, was only observed in 12% of the patients [11]. On the other hand, this study showed that complete response, defined by the most stringent criterion #1, was observed in 45.7% of the patients. From these results, we conclude that OA could be a treatment option for selected patients with REM-related OSA.

Several studies have reported the association between higher BMI and poor response to OA treatment [24, 25]. Therefore, we hypothesize that the difference in success rates between the study by Sutherland et al. and our own were mainly due to the higher BMI (30.0 ± 5.3 kg/m^2^) of the participants in the former study compared with our sample, as shown in Table 1. Sutherland et al. also reported that REM-related OSA showed a lower success rate than non-stage specific OSA [11]. In this study, no significant differences in success rate were found between the two groups for any criterion. In addition, the data in Table 4 indicates significant differences in the change of AHI, AI, maximum desaturation from baseline, and cumulative percentage of time spent at saturation below 90% (CT90) during REM and NREM sleep, before and after OA treatment, in both group. These data suggest similar efficacy of OA for REM-related OSA and non-stage-specific OSA in our non-obese sample.

Patients with REM-related OSA who did not respond to the OA treatment were analyzed in more detail. These patients had difficulty reducing their AHI during REM sleep, which decreased only from 36.2/h (24.4–44.2) to 23.9/h (16.7–37.2). Considering separately the apnea index (AI) and the hypopnea index (HI), we observed that the AI during REM sleep was sufficiently reduced from 26.3/h (6.1–33.0) to 7.1/h (1.4–14.9), but the HI was actually increased from 8.6/h (2.4–22.1) to 12.4/h (4.4–28.9). These results indicated that OA can prevent complete upper airway collapse, but partial collapse may retain during REM sleep.

Clinically, it is well known that obesity, by increasing upper airway vulnerability due to soft tissue crowdedness, leads to poor response to OA treatment [24]. Consistently with previous reports, BMI was a significant predictor of treatment outcome for OA when using 3 different criteria for treatment success as the response variable. Moreover, more than 10 cephalometric measurements have been reported as predictors of treatment outcome, but no cephalometric measurements were found to be significant predictors of treatment outcome in this study [13]. These results suggest that soft tissue crowdedness has more impact than craniofacial abnormalities on the effect of mandibular advancement in patients with REM-related OSA. ROC analysis showed that roughly the same cutoff values, as defined by the maximum Youden index, were obtained in this study for the three success criteria. These cutoff values could be used to select the patients for whom OA could be an effective treatment option for REM-related OSA.

Our study has several limitations. First, the cephalometry conducted in this study was performed under wakefulness and offers only a two-dimensional image in the upright position. This examination can sufficiently analyze the craniofacial bony enclosure, but does not reveal the characteristics of soft tissue inside the craniofacial bony enclosure during sleep. We plan to perform a similar study including craniofacial variables determined by magnetic imaging and computed tomography under sedation. A second limitation was the selection bias due to this study being conducted in a single facility. Third, the effects of unknown confounding factors, including the use of medication affecting REM sleep or decreasing muscle tone, could not be excluded because of the retrospective nature of this study. Further studies are needed to confirm the external validity of our results.

## Conclusion

No significant differences in success rate of OA treatment were founded between REM-related OSA and non-stage-specific OSA. BMI has more impact than craniofacial abnormality on treatment outcome in the patient with REM-related OSA. Cutoff values for BMI examined in this study could be used to select the patients for whom OA could be an effective treatment option. Further studies will be necessary to confirm the general applicability of our results.
